# Seroreactivity and Risk Factors Associated with *Coxiella burnetii* Infection among Cattle Slaughterhouse Workers in South Korea

**DOI:** 10.3390/ijerph15102264

**Published:** 2018-10-16

**Authors:** Ji-Hyuk Park, Seon Do Hwang, Dilaram Acharya, Seung Hun Lee, Kyu Jam Hwang, Seok-Ju Yoo, Kwan Lee

**Affiliations:** 1 Department of Preventive Medicine, College of Medicine, Dongguk University, Gyeongju 38066, Korea; skeyd@naver.com (J.-H.P.); dilaramacharya123@gmail.com (D.A.); medhippo@hanmail.net (S.-J.Y.); 2 Division of Zoonoses, Center for Immunology and Pathology, Korea National Institute of Health, Korea Centers for Disease Control and Prevention, Cheongju 28159, Korea; hwangsd@korea.kr (S.D.H.); lee5196@korea.kr (S.H.L.); kyuhwang@nih.go.kr (K.J.H.); 3 Division of Bacterial Diseases, Center for Laboratory Control of Infectious Diseases, Korea Centers for Disease Control and Prevention, Cheongju 28159, Korea; 4 Department of Community Medicine, Kathmandu University, Devdaha Medical College and Research Institute, Devdaha Municipality, Rupandehi 32900, Nepal; 5 Yeosu National Quarantine Office, Korea Centers for Disease Control and Prevention, Yeosu 59729, Korea; 6 Pathogen Resource TF, Center for Infectious Diseases, Korea National Institute of Health, Korea Centers for Disease Control and Prevention, Cheongju 28159, Korea

**Keywords:** *Coxiella burnetii*, slaughterhouse, serologic tests, risk factors, South Korea

## Abstract

Q fever, caused by *Coxiella burnetii*, is a zoonotic disease that is an occupational hazard to people who work in close contact with animals or their carcasses. A nationwide serologic study among cattle slaughterhouse workers who were presumed to be at risk of having *C. burnetii* infection in South Korea was performed to investigate the seroreactivity of *C. burnetii* infection and identify related risk factors. Out of 1017 cattle slaughterhouse workers in South Korea, 923 (90.8%) participated in this cross-sectional study. Samples were tested for immunoglobulin G (IgG) and M (IgM) antibodies against phase II *C. burnetii* via indirect immunofluorescence assay. The overall seroreactivity, defined as IgG or IgM antibody titer cutoffs ≥1:16, was 9.1% (84/923). Additionally, a significant association was found between the seroreactivity of *C. burnetii* infection and performing carcass evisceration work (odds ratio, 2.36; 95% confidence interval, 1.39–4.03) in multivariate analysis. To diminish *C. burnetii* infection, cattle slaughterhouse workers need to take precautions during the evisceration process.

## 1. Introduction

Q fever is a global zoonosis caused by the ubiquitous pathogen *Coxiella burnetii*, a microorganism that can withstand harsh physical conditions, including a hot and dry environment for a period ranging from months to years [1,2]. Domestic ruminants such as goat, sheep, and cattle are the primary reservoirs for this organism, although multiple vertebrate species have also been reported to be infected [3]. In animals, the majority of infections are asymptomatic, except for those affecting the reproductive system in females. However, infected ruminants can shed *C. burnetii* in feces, urine, milk, placental membranes, and amniotic fluids [4].

Clinical presentation of Q fever in humans is usually asymptomatic or as a mild disease with spontaneous recovery. However, a minority of patients may experience serious complications and even death [5]. The most common modes of human transmission occur either by direct inhalation of infective aerosol or by the inhalation of dust contaminated with infective materials such as feces, urine, and amniotic fluids [1]. In addition, the ingestion of raw milk and dairy products is a less commonly reported route of transmission [6]. Hence, individuals who have occupations with increased contact with animals or animal products, such as farmers, veterinarians, slaughterhouse workers, and laboratory workers, could have an increased risk for *C. burnetii* infection [7].

Several studies have investigated the *C. burnetii* serologic infection rate and the association between certain risk factors and *C. burnetii* infection among high risk groups. A study from Turkey [8] reported the highest seroprevalence among slaughterhouse workers, and two studies from Iran [9,10] reported a higher rate of seropositivity among slaughterhouse workers and butchers compared to other risk groups. Additionally, slaughterhouse workers who processed camel carcasses had an increased risk of developing *C. burnetii* seropositivity [9].

In South Korea, the majority of animals processed in slaughterhouses are cattle and pigs. A South Korean serologic study among slaughterhouse workers who were processing cattle, the primary reservoir of *C. burnetii*, reported *C. burnetii* seroreactivity of 11.3% [11]. However, the study did not examine the association between the types of cattle slaughter work or work hygiene-related factors. In the current study, we aimed to evaluate the seroreactivity and risk factors associated with *C. burnetii* infection among cattle slaughterhouse workers in South Korea.

## 2. Materials and Methods

### 2.1. Study Design

In 2012, there were a total of 1017 cattle slaughterhouse workers in 71 slaughterhouses according to data obtained from Ministry for Food, Agriculture, Forestry, and Fisheries in South Korea. We aimed to enroll all cattle slaughterhouse workers who were presumed to have *C. burnetii* infection in this cross-sectional study. Self-developed questionnaires were adapted from a previous study for cattle slaughterhouse workers [11]. In addition, several additional questions, based on available literature and the direct observation of several cattle slaughterhouses, were added to the questionnaires to solicit information on the types of slaughter work and work hygiene-related factors [12,13,14]. The types of cattle slaughter work were classified as follows: cattle transportation, stunning, bleeding, cutting (heads, front legs, and hind legs), skinning (manual and mechanical), chest opening, evisceration, body splitting, and carcass washing.

### 2.2. Data Collection

The cattle slaughterhouse workers were informed about the study and received the questionnaires prior to the slaughterhouse visits. Six study teams visited all the slaughterhouses during June 2012. The completed questionnaires were verified by researchers to ensure the quality of the data collected. Following this, the blood sample (10 mL) was obtained from the participants for *C. burnetii* antibody testing. Sera were kept at 4 °C and transported to the Korea Centers for Disease Control and Prevention within 24 h of collection.

### 2.3. Serologic Testing

An indirect immunofluorescence assay (IFA), the standard method of diagnosing Q fever [15], was used to detect immunoglobulin G (IgG) and immunoglobulin M (IgM) antibodies against phase II *C. burnetii* (Focus Diagnostics, Cyprus, CA, USA) according to manufacturer’s instruction. The samples were considered seroreactive when the IgG or IgM antibody titer cutoffs were ≥1:16, which are the same criteria used by a previous study performed among cattle slaughterhouse workers in South Korea [11].

### 2.4. Statistical Analysis

We conducted univariate logistic regression analysis to determine the factors associated with *C. burnetii* infection among slaughterhouse workers. Subsequently, variables determined to be important (*p* < 0.10) were incorporated into the multivariate logistic regression analysis to calculate odds ratios (ORs) with 95% confidence intervals (CIs). The statistical significance was set at *p* < 0.05. The data were analyzed using SPSS Version 17.0 (SPSS Inc., Chicago, IL, USA).

### 2.5. Ethical Considerations

The Institutional Review Board of Dongguk University Gyeongju Hospital reviewed and approved this study (approval number: 12-033). Informed consent was obtained from all participants prior to enrollment in this study.

## 3. Results

Out of 1017 slaughterhouse workers, 923 (90.8%) participated in this study. The participants consisted of 865 men (93.7%) and 58 women (6.3%), with a median age of 51 years. The median duration of work was 11 years, and the median slaughterhouse scale was 40 cattle per day.

### 3.1. Serologic Profiles and Seroreactivity of C. burnetii 

The titer cutoffs of phase II IgG antibodies against *C. burnetii* ranged from <1:16 to 1:2048; 84 samples (9.1%) had IgG titers ≥1:16. The titer cutoffs of phase II IgM antibodies against *C. burnetii* ranged from <1:16 to 1:32; two samples (0.2%) had IgM titers ≥1:16, both of which had IgG titers of 1:64. Overall, 84 out of 923 participants (9.1%) were seroreactive for *C. burnetii* (Table 1).

### 3.2. Univariate Analysis of C. burnetii Seroreactivity and Potential Risk Factors

Increased age (≥60 years) was associated with a higher risk of *C. burnetii* infection compared to younger individuals (<45 years) (*p* = 0.091). Additionally, a lower level of education was associated with a higher risk of *C. burnetii* infection (*p* = 0.071). However, sex, duration of work, slaughterhouse scale, and consumption of raw milk were not associated with *C. burnetii* seroreactivity (Table 2). Among the types of slaughter work, cattle slaughterhouse workers who performed carcass evisceration had a higher risk of *C. burnetii* infection (*p* = 0.001). Other types of slaughter work were not associated with *C. burnetii* seroreactivity (Table 3). The questionnaires also solicited information about the use of personal protective equipment (e.g., glasses, masks, gloves, aprons, and boots), the habit of showering after work, contact with blood or feces, and the presence of wounds on skin. Only oral exposure to blood (≥ once a week) was associated with a higher risk of *C. burnetii* infection (*p* = 0.072, Table 4).

### 3.3. Multivariate Analysis of C. burnetii Seroreactivity and Important Risk Factors

The following factors identified as being of importance (*p* < 0.10) in the univariate analysis were included in the multivariate model: age, education, performing carcass evisceration, and oral exposure to blood. Of these variables, performing carcass evisceration (OR, 2.36; 95% CI, 1.39–4.03) was significantly associated with a higher risk of *C. burnetii* infection. However, oral exposure to blood had a higher risk of *C. burnetii* infection without statistical significance (OR, 1.49; 95% CI, 0.93–2.37, Table 5).

## 4. Discussion

In this study, we determined that *C. burnetii* seroreactivity was 9.1% (84/923), and that seroprevalence (phase II IgG or IgM titer ≥1:64) was 5.2% (48/923) among cattle slaughterhouse workers. Slaughterhouse workers are one of the groups that are occupationally exposed to *C. burnetii*, and their seroprevalence was considerably higher than that among asymptomatic people in a rural area in South Korea (1.5%) [16]. According to the previous South Korean study conducted among cattle slaughterhouse workers in 2007, the seroreactivity and seroprevalence against *C. burnetii* were 11.3% and 4.0%, respectively [11] which is consistent with the current study’s findings. This serologic trend corresponds with the few Q fever cases in South Korea during 2007–2012 (approximately 10 cases annually) [17].

An Iranian serologic study of *C. burnetii* infection tested by IFA reported a seroprevalence (phase II IgG titer ≥1:64) of 46.5% among slaughterhouse workers and butchers [18], while another similar study which tested by enzyme-linked immunosorbent assay (ELISA) in the southeast of Iran identified a seroprevalence of 68.0% among slaughterhouse workers [19]. An Italian study performed among slaughterhouse workers produced a comparatively high seroprevalence of 73.7%, tested by ELISA [20]. All these three studies reported a higher prevalence of *C. burnetii* infection among slaughterhouse workers than we found in South Korea. The variation could to attributable to the differences in serologic testing methodology, the *C. burnetii* infection status of the slaughtered animals, the level of exposure to risk factors and whether or not effective protective measures were used during the slaughtering process. In summary, *C. burnetii* infection among slaughterhouse workers in South Korea was found to be lower than among their counterparts in other countries. Interestingly, a study of the seroprevalence of *C. burnetii* infection among cattle in South Korea reported the lowest seroprevalence among Korean native cattle, which comprised the main slaughtering cattle in 2012 [13,21].

We found that cattle slaughterhouse workers who were involved in carcass evisceration yielded significantly higher odds of having *C. burnetii* infection. Infected cattle shed a large amount of *C. burnetii* in feces, urine, milk, and vaginal mucus [15]. During the evisceration process, leakage of feces in the digestive tract and urine and vaginal mucus in the urogenital tract may occur. The increased concentration of *C. burnetii* in the air around the evisceration work environment might facilitate the spread of infection. However, manual skinning, including mammary gland removal, was not significantly associated with *C. burnetii* infection. Considering that cattle are not usually slaughtered during the peripartum period, when cattle produce milk, slaughterhouse workers performing manual skinning are rarely exposed to milk. Other slaughtering processes that could create aerosols through the use of mechanical devices (cutting, mechanical skinning, chest opening, and body splitting) were also not significantly associated with *C. burnetii* infection. This might be influenced by discordance between the shedding sites of *C. burnetii* and the cutting sites of those slaughtering processes.

Our multivariate analysis also revealed that oral exposure to blood was not significantly associated with *C. burnetii* infection. Similarly, a study among slaughterhouse workers and butchers in Iran reported that the splashing of animal secretions on the face or body was not significantly associated with *C. burnetii* infection [9]. In addition, the use of personal protective equipment, including wearing a protective mask, was not significantly associated with a lower risk of *C. burnetii* infection. However, protective masks (N-95 or above) have been identified as a protective factor for *C. burnetii* infection [7,22]. We assume that slaughterhouse workers in South Korea might use insufficiently protective masks (such as cloth masks or face masks) rather than N-95 masks. Slaughterhouse workers over 60 years of age were not at increased risk for seroreactivity in our multivariate analysis. Other serologic studies among slaughterhouse workers also reported no association between older age and *C. burnetii* infection [9,19].

This was a nationwide cross-sectional study of *C. burnetii* infection among cattle slaughterhouse workers in South Korea; most of the study participants participated in the study, giving a high response rate of 90.8%. However, the study should address some specific limitations. First, we only collected serum samples during summer. The rate of *C. burnetii* infection in cattle might be affected by seasonal factors [15], and this could influence the seroreactivity rate among cattle slaughterhouse workers. Second, although we investigated various slaughtering work-related risk factors, some other lifestyle-related potential risk factors—for instance, exposure to wildlife and other sources of *C. burnetii* infection—could not be ruled out. Third, we could not investigate the *C. burnetii* infection status of the slaughtered cattle, which might influence the seroreactivity of cattle slaughterhouse workers. In addition, our result demonstrated that performing carcass evisceration work was a potential risk factor of *C. burnetii* infection in general, rather than being cattle-specific. However, this study explored an important area of research that could help develop evidence-based preventive strategies to reduce the burden of Q fever.

## 5. Conclusions

In conclusion, our study investigated the *C. burnetii* seroreactivity rate of 9.1% among cattle slaughterhouse workers in South Korea. We identified performing carcass evisceration work as a risk factor for *C. burnetii* infection. To diminish *C. burnetii* infection, slaughterhouse workers should take precautions during the evisceration process, and furthermore, the working environment of slaughterhouses during the evisceration process might need to be improved.

## Figures and Tables

**Table 1:** Serologic results for *Coxiella burnetii* phase II antigen among cattle slaughterhouse workers in South Korea.

Titer	IgG		IgM	
	No.	%	No.	%
<1:16	839	90.9	921	99.8
1:16	18	2.0	1	0.1
1:32	18	2.0	1	0.1
1:64	20	2.2	0	0.0
1:128	15	1.6	0	0.0
≥1:256	13	1.4	0	0.0
Total	923	100.0	100.0	100.0

Ig, immunoglobulin.

**Table 2:** Association of demographic characteristics with *Coxiella burnetii* seroreactivity among cattle slaughterhouse workers in South Korea.

	Total	Seroreactivity No. (%)	OR (95% CI)	*p* Value ^a^
Sex				
Male	865	82 (9.5)	2.93 (0.70–12.24)	0.140
Female	58	2 (3.4)	Reference	
Age (years)				
<45	272	20 (7.4)	Reference	
45–59	506	46 (9.1)	1.26 (0.73–2.18)	0.408
≥60	145	18 (12.4)	1.79 (0.91–3.50)	0.091
Duration of work (years)				
<15	543	47 (8.7)	Reference	
15–29	293	25 (8.5)	0.98 (0.59–1.64)	0.952
≥30	77	9 (11.7)	1.40 (0.66–2.98)	0.387
Region				
Northern	200	13 (6.5)	Reference	
Central	463	43 (9.3)	1.47 (0.77–2.80)	0.239
Southern	260	28 (10.8)	1.74 (0.87–3.45)	0.115
Slaughterhouse scale (cattle per day)				
<30	292	28 (9.6)	Reference	
30–59	315	24 (7.6)	0.78 (0.44–1.38)	0.387
≥60	316	32 (10.1)	1.06 (0.62–1.81)	0.824
Education				
Middle school or less	440	48 (10.9)	1.52 (0.96–2.39)	0.071
High school or more	482	36 (7.5)	Reference	
Consumption of raw beef				
Yes (≥once a week)	453	40 (8.8)	0.94 (0.60–1.47)	0.771
No	469	44 (9.4)	Reference	
Consumption of raw by-products ^b^				
Yes (≥once a week)	319	27 (8.5)	0.89 (0.55–1.43)	0.620
No	603	57 (9.5)	Reference	
Consumption of raw milk				
Yes (within a year)	16	2 (12.5)	1.43 (0.32–6.38)	0.643
No	900	82 (9.1)	Reference	
Breeding cattle				
Yes	42	1 (2.4)	0.24 (0.03–1.77)	0.162
No	857	79 (9.2)	Reference	

OR, odds ratio; CI, confidence interval. ^a^ Univariate logistic regression was applied. ^b^ Raw by-products refer to raw liver or stomach tissue.

**Table 3:** Association of work types with *Coxiella burnetii* seroreactivity among cattle slaughterhouse workers in South Korea.

	Total	Seroreactivity No. (%)	OR (95% CI)	*p* Value ^a^
Cattle transportation				
Yes	141	9 (6.4)	0.64 (0.31–1.32)	0.226
No	782	75 (9.6)	Reference	
Stunning				
Yes	72	7 (9.7)	1.08 (0.48–2.44)	0.849
No	851	77 (9.0)	Reference	
Bleeding				
Yes	89	9 (10.1)	1.14 (0.55–2.36)	0.727
No	834	75 (9.0)	Reference	
Cutting of heads				
Yes	122	14 (11.5)	1.35 (0.74–2.49)	0.329
No	801	70 (8.7)	Reference	
Cutting of front legs				
Yes	82	7 (8.5)	0.93 (0.41–2.08)	0.852
No	841	77 (9.2)	Reference	
Cutting of hind legs				
Yes	115	15 (13.0)	1.61 (0.89–2.92)	0.119
No	808	69 (8.5)	Reference	
Manual skinning				
Yes	133	13 (9.8)	1.10 (0.59–2.04)	0.770
No	790	71 (9.0)	Reference	
Mechanical skinning				
Yes	137	11 (8.0)	0.85 (0.44–1.65)	0.637
No	786	73 (9.3)	Reference	
Chest opening				
Yes	84	9 (10.7)	1.22 (0.59–2.54)	0.590
No	839	75 (8.9)	Reference	
Evisceration				
Yes	129	22 (17.1)	2.43 (1.43–4.11)	0.001
No	794	62 (7.8)	Reference	
Body splitting				
Yes	83	7 (8.4)	0.91 (0.41–2.05)	0.825
No	840	77 (9.2)	Reference	
Carcass washing				
Yes	85	7 (8.2)	0.89 (0.40–1.99)	0.771
No	838	77 (9.2)	Reference	

OR, odds ratio; CI, confidence interval. ^a^ Univariate logistic regression was applied.

**Table 4:** Association of work hygiene-related factors with *Coxiella burnetii* seroreactivity among cattle slaughterhouse workers in South Korea.

	Total	Seroreactivity No. (%)	OR (95% CI)	*p* Value ^a^
Wearing protective glasses				
Always	224	21 (9.4)	1.02 (0.61–1.72)	0.932
Sometimes/rarely	686	63 (9.2)	Reference	
Wearing a protective mask				
Always	523	51 (9.8)	1.17 (0.74–1.85)	0.497
Sometimes/rarely	391	33 (8.4)	Reference	
Wearing protective gloves				
Always	730	68 (9.3)	1.05 (0.59–1.86)	0.860
Sometimes/rarely	180	16 (8.9)	Reference	
Wearing a protective apron				
Always	824	78 (9.5)	1.53 (0.65–3.62)	0.329
Sometimes/rarely	94	6 (6.4)	Reference	
Wearing protective boots				
Always	878	81 (9.2)	1.29 (0.39–4.26)	0.679
Sometimes/rarely	41	3 (7.3)	Reference	
Taking a shower after work				
Always	894	82 (9.2)	1.31 (0.31–5.63)	0.714
Sometimes/rarely	28	2 (7.1)	Reference	
Contact with blood around the mouth				
Yes (≥once a week)	473	51 (10.8)	1.52 (0.96–2.41)	0.072
No	449	33 (7.3)	Reference	
Contact with blood around the body				
Yes (≥once a week)	642	62 (9.7)	1.25 (0.75–2.08)	0.391
No	279	22 (7.9)	Reference	
Contact with feces/urine around the mouth or body				
Yes (≥once a week)	526	51 (9.7)	1.17 (0.74–1.86)	0.492
No	394	33 (8.4)	Reference	
Presence of wound on skin				
Yes (within a year)	134	12 (9.0)	0.97 (0.51–1.84)	0.929
No	783	72 (9.2)	Reference	

OR, odds ratio; CI, confidence interval. ^a^ Univariate logistic regression was applied.

**Table 5:** Multivariate logistic regression analysis of important variables (*p* < 0.10) associated with *Coxiella burnetii* seroreactivity among cattle slaughterhouse workers in South Korea.

	OR (95% CI)	*p* Value
Age (years)		
<45	Reference	
45–59	1.06 (0.57–1.98)	0.852
≥60	1.59 (0.73–3.47)	0.248
Education		
Middle school or less	1.33 (0.78–2.29)	0.294
High school or more	Reference	
Evisceration		
Yes	2.36 (1.39–4.03)	0.002
No	Reference	
Contact with blood around the mouth		
Yes (≥ once a week)	1.49 (0.93–2.37)	0.095
No	Reference	

OR, odds ratio; CI, confidence interval.
